# Uncovering serum placental-related non-coding RNAs as possible biomarkers of preeclampsia risk, onset and severity revealed MALAT-1, miR-363 and miR-17

**DOI:** 10.1038/s41598-022-05119-9

**Published:** 2022-01-24

**Authors:** Samy A. Abdelazim, Olfat G. Shaker, Yehya Aly Hussein Aly, Mahmoud A. Senousy

**Affiliations:** 1 Biochemistry Department, Faculty of Pharmacy, Cairo University, Cairo, 11562 Egypt; 2 Medical Biochemistry and Molecular Biology Department, Faculty of Medicine, Cairo University, Cairo, Egypt; 3 Pharmacist at Kasr Al-Ainy Hospital, Cairo University, Cairo, Egypt

**Keywords:** Molecular biology, Biomarkers, Diseases, Molecular medicine

## Abstract

New predictors that could boost early detection of preeclampsia (PE) and prognosticate its severity are urgently needed. We examined serum miR-17, miR-363, MALAT-1 and HOTAIR as potential biomarkers of PE risk, onset and severity. This prospective study included 160 pregnant females; 82 PE cases and 78 healthy pregnancies. Serum samples were collected between 20 to 40 weeks of gestation. Early-onset PE was defined as developing clinical manifestations at ≤ 34 gestational weeks. Severe PE was defined as systolic blood pressure ≥ 160 mmHg and/or diastolic blood pressure ≥ 110 mmHg and proteinuria (≥ 2 g/24 h or ≥ 2+ dipstick). Selection of PE-related non-coding RNAs and functional target gene analysis were conducted using bioinformatics analysis. Expression profiles were assessed by RT-qPCR. Serum miR-363 and MALAT-1 were downregulated, meanwhile miR-17 was upregulated, and HOTAIR was not significantly altered in PE compared with healthy pregnancies. miR-17 was elevated while miR-363 and MALAT-1 were reduced in severe versus mild PE. miR-363 was lower in early-onset versus late-onset PE. MALAT-1, miR-17 and miR-363 showed diagnostic potential and discriminated severe PE, whereas miR-363 distinguished early-onset PE in the receiver-operating-characteristic analysis. miR-363 and MALAT-1 were significantly associated with early and severe PE, respectively in multivariate logistic analysis. In PE, miR-17 and MALAT-1 were significantly correlated with gestational age (r = − 0.328 and r = 0.322, respectively) and albuminuria (r = 0.312, and r = − 0.35, respectively). We constructed the MALAT-1, miR-363, and miR-17-related protein–protein interaction networks linked to PE. Serum miR-17, miR-363 and MALAT-1 could have utility as new biomarkers of PE diagnosis. miR-363 may be associated with early-onset PE and MALAT-1 downregulation correlates with PE severity.

## Introduction

Preeclampsia (PE) is a multi-systemic pregnancy disorder that globally affects 2–10% of pregnancies, and currently is among the most common causes of maternal death^1^. PE mostly uncovers pre-existing endothelial dysfunction, metabolic and vascular diseases^2^, which necessitate long-term monitoring of women with prior exposure to PE, preterm birth, or delivery of an infant small for gestational age^3^. Thus, new biomarkers of PE risk, onset and severity are urgently needed.

The pathogenesis of PE includes inadequate trophoblastic invasion, developmental abnormalities of placental vasculature and placental underperfusion, however the exact pathophysiological mechanism remains unknown^4^. Thereby, recognizing the mechanistic insights underlying trophoblastic behavior and pathology in PE will clear up new biomarkers for its diagnosis and prognosis as well as novel therapeutic targets.

Non-coding RNAs (ncRNAs), including microRNAs (miRNAs; 18–22 nucleotides) and long ncRNAs (lncRNAs; > 200 nucleotides) are involved in several cellular paradigms in the placenta, including trophoblastic invasion, cell proliferation and endothelial function^5,6^. lncRNAs also act as competing endogenous RNAs (ceRNAs) for miRNAs, and this crosstalk was linked to PE pathology^6^. Deregulated miRNAs and lncRNAs are released into the circulation from the defective placenta in PE and have come out as potential biomarkers for its early screening and diagnosis and as novel targets for its prevention and treatment^7–9^.

Although several reports revealed the probable usefulness of measuring certain ncRNAs as circulating biomarkers of PE^7–9^, a study by luque et al. concluded that measurement of maternal serum miRNA at the first-trimester of pregnancy lacks any predictive value for early PE^10^. For this divergence, more investigations are needed to shed light on the precise role of PE-related ncRNAs and their possible cross-interaction in predicting its onset and severity. Accordingly, we have conducted a systematic bioinformatics approach to select PE-associated lncRNAs and miRNAs, and then examined candidate lncRNAs-miRNAs predicted and reported interactions. Herein, the lncRNAs metastasis associated lung adenocarcinoma transcript-1 (MALAT-1) and homeobox transcript antisense RNA (HOTAIR) as well as miR-17 and miR-363 were selected.

miR-17, a member of the miR-17-92 cluster, is a placental-specific miRNA which is largely located in various subtypes of trophoblasts, regulating important trophoblastic cell events, including differentiation, angiogenesis and apoptosis^9^. In particular, miR-17 regulates multiple steps during placental angiogenesis and its deregulation causes placental developmental defects^11^. Another placental-related miRNA is miR-363, a member of miR-106a-363 cluster, which is highly expressed during placental development in rapidly proliferating cytotrophoblasts (CTB), the most metabolically active cells in the placenta. Members of miR-17-92 and miR-106a-363 clusters are MYC-responsive targets, an important regulator of early placentation^12^, and were established to target several genes that are involved in CTB proliferation, differentiation, angiogenesis and metabolic reprogramming^12^. The lncRNA MALAT-1 regulates trophoblast invasion during placental development; its knockdown impaired trophoblast invasion and proliferation, suppressed the cell cycle progression and promoted apoptosis^13^. HOTAIR lncRNA regulates proliferation, invasion and apoptosis of placental trophoblasts; its overexpression decreased their invasive capacity and increased cell apoptosis^14^. However, the exact role of these ncRNAs, their crosstalk and clinical relevance in PE are not fully elucidated.

Thereby, the present study investigated the circulating expression profiles of miR-17, miR-363, MALAT-1 and HOTAIR in sera of PE patients and normal pregnancies, and evaluated their potential value as biomarkers of PE risk, onset and severity. We also explored the correlation between these ncRNAs and clinicopahological data of PE patients. Target gene analysis was also conducted using online softwares and databases to functionally relate these ncRNAs to PE pathogenesis through constructing the target protein–protein interaction (PPI) networks linked to PE.

## Results

### PE characteristics

Both PE patients and healthy pregnancies were comparable in terms of age, smoking status, body mass index (BMI), parity, abortion, gravidity, fasting blood sugar, liver function tests, serum urea and hematological parameters (*P* > 0.05). Patients with PE exhibited significantly higher systolic blood pressure (SBP), diastolic blood pressure (DBP), mean arterial pressure (MAP), C-reactive protein (CRP) (*P* < 0.0001 for each), serum creatinine and uric acid levels (*P* = 0.0003 for each). PE patients showed low amniotic fluid (oligohydraminos), intrauterine growth restriction (IUGR), and abnormal Doppler (*P* < 0.0001 for each) more frequently than the control group. Moreover, PE patients had lower gestational age (GA) (*P* = 0.045) and fetal birth weight (FBW) (*P* < 0.0001), in addition to, more frequent caesarean delivery (*P* = 0.003) (Table 1).

**Table 1:** Characteristics of PE patients and healthy pregnancies.

Parameters	Controls (n = 78)	Preeclampsia (n = 82)	*P value*
Maternal age (years), range	31 ± 6.55 (18–41)	29.73 ± 6.83 (18–42)	0.23
Body mass index (BMI) (kg/m^2^)	32.36 ± 4.7	31.2 ± 5.26	0.14
Parity (n)	1.64 ± 1.78	1.73 ± 1.62	0.73
Abortion (n)	0.82 ± 1.45	0.707 ± 1.44	0.62
Gravidity (n)	2.46 ± 2.47	2.44 ± 2.41	0.95
GA (weeks)	37.31 ± 1.53	36.65 ± 2.47	0.045*
FBW (kg)	3.28 ± 0.38	2.84 ± 0.49	< 0.0001*
SBP (mmHg)	116.8 ± 12.87	164.4 ± 17.47	< 0.0001*
DBP (mmHg)	72.31 ± 7.01	108.3 ± 10.98	< 0.0001*
MAP (mmHg)	94.55 ± 8.25	136.3 ± 12.85	< 0.0001*
CRP (mg/L)	6 (3–30)	27 (5–56.25)	< 0.0001*
Hb (g/dL)	10.74 ± 1.24	10.74 ± 1.19	0.99
TLC * 1000 (cells/mm^3^)	8.12 ± 2.59	8.23 ± 3	0.8
Platelet count * 1000 (cells/mm^3^)	304.26 ± 80.26	286.78 ± 95	0.21
Fasting plasma glucose (mg/dL)	82.85 ± 13.75	81.24 ± 13.18	0.45
AST (U/L)	18.46 ± 8.95	21.59 ± 10.5	0.06
ALT (U/L)	18.36 ± 10.09	20.66 ± 9.06	0.14
ALP (U/L)	73.82 ± 2.11	74.85 ± 5.94	0.15
Albumin (g/dL)	3.19 ± 0.27	3.12 ± 0.38	0.17
Total bilirubin (mg/dL)	0.6 ± 0.25	0.53 ± 0.26	0.097
Prothrombin time (s)	12.66 ± 0.79	12.62 ± 0.69	0.75
Urea (mg/dL)	26.23 ± 10.14	25.25 ± 9.9	0.53
Creatinine (mg/dL)	0.68 ± 0.197	0.81 ± 0.246	0.0003*
Uric acid (mg/dL)	3.55 ± 0.41	3.89 ± 0.69	0.0003*
**Smoking, n (%)**			0.1
Yes	27 (34.6)	39 (47.5)	
No	51 (65.4)	43 (52.5)	
**Amniotic fluid, n (%)**			< 0.0001*
Normal	72 (92.3)	36 (44)	
Low	6 (7.7)	46 (56)	
**Doppler, n (%)**			< 0.0001*
Normal	72 (92.3)	48 (58.5)	
Abnormal	6 (7.7)	34 (41.5)	
**IUGR, n (%)**			< 0.0001*
No	72 (92.3)	42 (51.2)	
Yes	6 (7.7)	40 (48.8)	
**MOD, n (%)**			0.003*
VD	31 (40)	15 (18.3)	
CS	47 (60)	67 (81.7)	
**Albuminuria, n (%)**			Nil
4+	Nil	18 (22)	
3+		22 (27)	
2+		24 (29)	
1+		18 (22)	

Results are presented as mean ± SD, median (25%–75% percentiles) or number (percentage). ALP, alkaline phosphatase; ALT, alanine transaminase; AST, aspartate transaminase; BMI, body mass index; CRP, C-reactive protein; CS, caesarean section; DBP, diastolic blood pressure; FBW, fetal birth weight; GA, gestational age; Hb, hemoglobin; IUGR, intrauterine growth restriction; MAP, mean arterial pressure; MOD, mode of delivery; SBP, systolic blood pressure; TLC, total leukocyte count; VD, vaginal delivery. *Statistically significant, *P* < 0.05. Blood pressure was measured at admission for controls and PE patients. GA was measured at admission using ultrasound device. IUGR was defined as weight below the 10th percentile for the GA.

Thirty-four percent of patients were diagnosed with mild PE while the rest had severe PE. Thirty-nine percent of PE patients were early-onset cases while the others were late-onset cases.

### Expression of studied ncRNAs in PE versus healthy pregnancies

**Figure 1:**
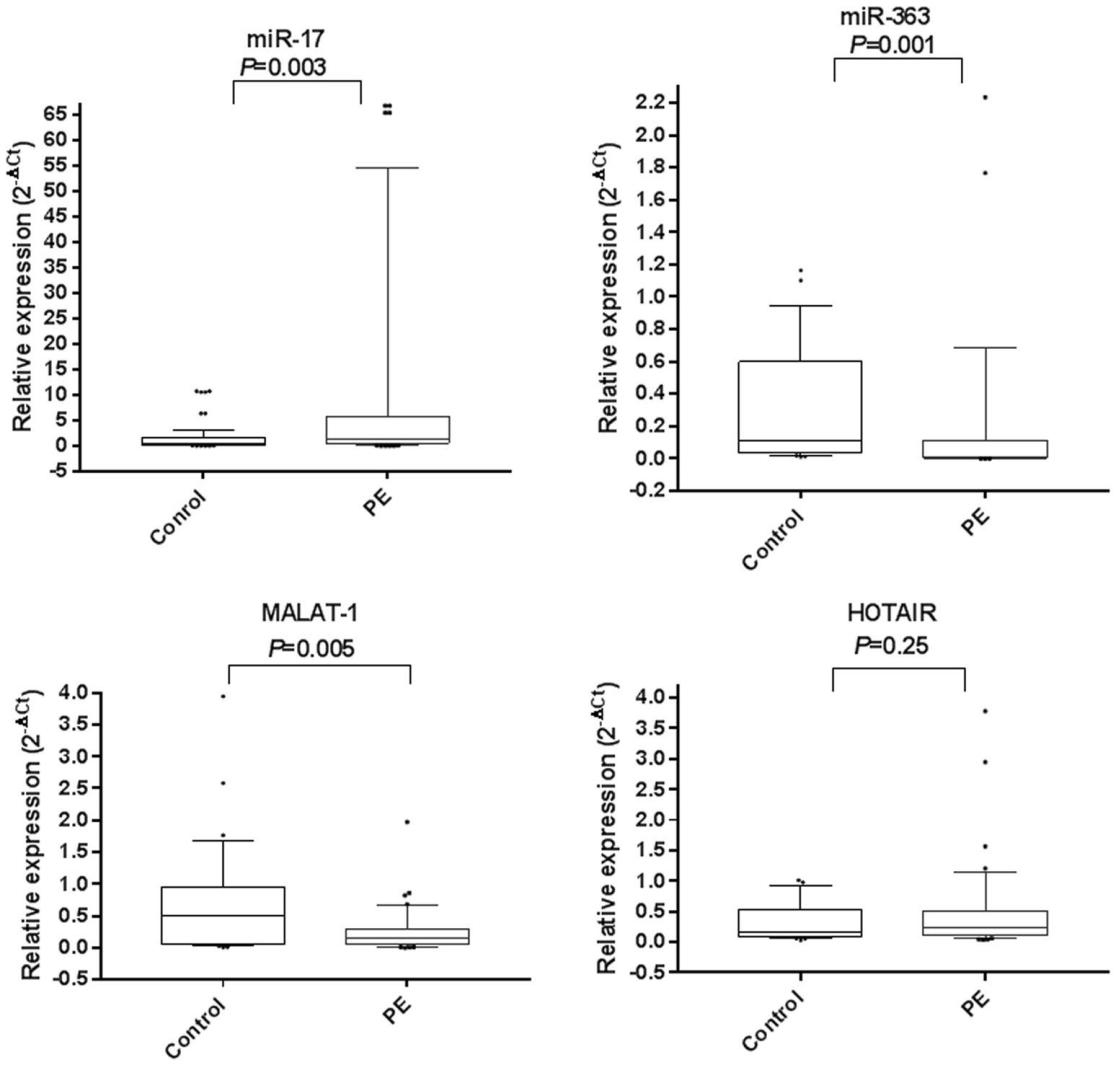
Serum expression profiles of miR-17, miR-363, MALAT-1 and HOTAIR in PE patients. The box represents the 25–75% percentiles; the line inside the box represents the median and the upper and lower lines representing the 10%-90% percentiles of relative gene expression levels (2^−∆Ct^) of studied parameters in PE (n = 82) compared to healthy pregnancies (n = 78).

The studied ncRNAs were expressed in sera of PE cases and healthy pregnancies with varying levels (Fig. 1). Notably, serum miR-17 was upregulated by a median 3.5-fold (*P* = 0.003), while serum miR-363 and MALAT-1 were downregulated by median 4 and 2.1-fold, respectively (*P* = 0.001 and *P* = 0.005, respectively) in PE patients compared to controls. On the other hand, serum HOTAIR expression levels was not changed between PE patients and healthy pregnancies (*P* = 0.25) (Fig. 1 and Table 2).

**Table 2:** Fold change of serum miR-17, miR-363, MALAT-1 and HOTAIR expression levels in PE patients regarding risk, severity and onset.

Parameter	PE (n = 82) compared to healthy pregnancies (n = 78)	*P* value	
miR-17	3.5 (0.95–27.74)	0.003*	
miR-363	0.25 (0.026–4.287)	0.001*	
HOTAIR	0.98 (0.42–2.1)	0.25	
MALAT-1	0.48 (0.15–0.91)	0.005*	

	Mild PE (n = 28)	Severe PE (n = 54)	*P* value
miR-17	0.94 (0.427–9.6)	3.4 (2.37–14.3)	0.002*
miR-363	1.53 (0.186–4.417)	0.027 (0.01–0.23)	0.005*
HOTAIR	1.53 (0.663–4)	1.715 (0.69–3.07)	0.67
MALAT-1	1.078 (0.436–2.38)	0.238 (0.064–0.64)	0.0004*

	Early-onset PE (n = 32)	Late-onset PE (n = 50)	*P* value
miR-17	2.63 (0.55–7.4)	2.81 (1.93–12.3)	0.58
miR-363	0.018 (0.001–0.326)	0.158 (0.028–2.23)	0.001*
HOTAIR	1.27 (0.66–3.18)	1.97 (0.69–3.31)	0.33
MALAT-1	0.3 (0.112–0.5)	0.24 (0.064–0.71)	0.76

	Early-onset PE (n = 32) compared to early controls (n = 40)	*P* value	
miR-17	1.97 (0.41–5.55)	0.2	
miR-363	0.025 (0.003–0.44)	< 0.0001*	
HOTAIR	1.625 (0.84–4.08)	0.37	
MALAT-1	0.46 (0.17–0.76)	0.37	

	Late-onset PE (n = 50) compared to late controls (n = 38)	*P* value	
miR-17	5.46 (3.97–25.28)	0.0003*	
miR-363	0.68 (0.12–9.65)	0.8	
HOTAIR	1.91 (0.67–3.2)	0.68	
MALAT-1	0.22 (0.057–0.63)	< 0.0001*	

Data are presented as median (25%–75% percentiles) of the fold change data. The Mann-Whitney U test was used for data analysis. *indicates statistical significance (*P* < 0.05). Fold change was calculated using 2^–∆∆Ct^ relative to the corresponding control group.

Bold values indicates statistical significance.

### Expression of studied ncRNAs in PE patients regarding PE severity

Serum miR-17 levels were elevated in severe PE compared to mild cases (*P* = 0.002). Conversely, serum miR-363 (*P* = 0.005) and MALAT-1 (*P* = 0.0004) levels were reduced in severe PE compared to mild disease. Again, no changes were observed in serum HOTAIR levels between the two groups (*P* = 0.67) (Table 2).

### Expression of studied ncRNAs in PE patients regarding PE onset

Only serum levels of miR-363 showed differential expression between early-onset and late-onset PE cases, with markedly lower levels in the early-onset PE group (*P* = 0.001) (Table 2). To further examine the role of studied ncRNAs with the risk of early- or late-onset PE, we conducted a stratification analysis comparing early- or late-onset PE vs early or late controls (with matched gestational weeks at delivery, *P* > 0.05), respectively (Table 2). We recorded a marked downregulation of serum miR-363 in early-onset PE compared with early controls (*P* < 0.0001), while levels of other studied ncRNAs were not significantly different in the same comparison. On the other hand, we observed serum miR-17 upregulation (*P* = 0.0003) as well as MALAT-1 downregulation (*P* < 0.0001) in the late-onset PE group when compared with the late controls, while miR-363 and HOTAIR levels were comparable among the two groups (*P* = 0.8 and 0.68, respectively).

### Correlation of studied ncRNAs with each other in PE patients

In the whole PE group, we recorded many significant correlations between serum levels of the investigated ncRNAs where MALAT-1 was positively correlated with both miR-17 (r = 0.401, *P* = 0.017) and HOTAIR (r = 0.453, *P* = 0.0004) expression levels. Also, there was a positive correlation between miR-17 and miR-363 (r = 0.273, *P* = 0.015) (Table 3).

**Table 3:** Correlation between studied ncRNAs with each other and with clinicopathological data in PE patients.

Data	miR-17	miR-363	HOTAIR	MALAT-1
**Maternal age**				
r	− 0.25	− 0.034	− 0.232	**− 0.341**
*P*	0.115	0.832	0.144	0.029*
**BMI**				
r	**0.43**	**− 0.315**	0.119	0.074
*P*	0.002*	0.045*	0.457	0.644
**Abortion**				
r	0.058	0.113	0.063	− 0.182
*P*	0.719	0.482	0.693	0.256
**GA**				
r	**− 0.328**	**0.412**	− 0.066	**0.322**
*P*	0.036*	0.006*	0.682	0.04*
**Smoking**				
r	− 0.136	− 0.108	− 0.045	− 0.141
*P*	0.397	0.501	0.778	0.378
**SBP**				
r	0.034	0.126	0.06	0.163
*P*	0.833	0.432	0.711	0.308
**DBP**				
r	0.039	0.09	0.08	0.099
*P*	0.811	0.575	0.621	0.539
**MAP**				
r	0.019	0.164	0.072	0.086
*P*	0.907	0.307	0.657	0.594
**CRP**				
r	− 0.134	0.078	0.028	− 0.155
*P*	0.403	0.629	0.864	0.332
**Albuminuria**				
r	**0.312**	− 0.029	**0.274**	**− 0.35**
*P*	0.04*	0.856	0.039*	0.025*
**Uric acid**				
r	− 0.204	0.226	− 0.083	0.014
*P*	0.2	0.156	0.606	0.929
**FBW**				
r	**− 0.324**	0.128	0.15	− 0.018
*P*	0.039*	0.424	0.348	0.912
**IUGR**				
r	0.3	0.18	− 0.134	0.049
*P*	0.04*	0.103	0.403	0.759
**Abnormal Doppler**				
r	**0.234**	**− 0.26**	− 0.295	− 0.155
*P*	0.046*	0.04*	0.061	0.334
**Low amniotic fluid**				
r	**0.245**	0.031	− 0.16	0.042
*P*	0.044*	0.847	0.318	0.797
**MOD**				
r	**0.441**	0.151	0.079	0.23
*P*	0.004*	0.347	0.622	0.148
**miR-17**				
r	**–**	**0.273**	0.232	**0.401**
*P*		0.015*	0.145	0.017*
**miR-363**				
r	**0.273**	–	0.121	− 0.112
*P*	0.015*		0.518	0.745
**HOTAIR**				
r	0.232	0.121	–	**0.453**
*p*	0.145	0.518		0.0004*
**MALAT-1**				
r	**0.401**	− 0.112	**0.453**	–
*P*	0.017*	0.745	0.0004*	

*P < 0.05: statistically significant. BMI, body mass index; CRP, C-reactive protein; DBP, diastolic blood pressure; FBW, fetal birth weight; GA, gestational age; HOTAIR, homeobox transcript antisense RNA; IUGR, intrauterine growth restriction; MALAT-1, metastasis associated lung adenocarcinoma transcript 1; MAP, mean arterial pressure; MOD, mode of delivery; SBP, systolic blood pressure.

Bold values indicate statistical significance.

### Correlation of studied ncRNAs with clinicopathological data of PE patients

In the whole PE group, we noticed many significant correlations between serum levels of the investigated ncRNAs and clinicopathological data (Table 3). MiR-17 showed positive correlations with BMI (r = 0.43, *P* = 0.002), IUGR (r = 0.3, *P* = 0.04), abnormal Doppler (r = 0.234, *P* = 0.046), low amniotic fluid (r = 0.245, *P* = 0.044), mode of delivery (MOD) (r = 0.441, *P* = 0.004) and albuminuria (r = 0.312, *P* = 0.04), while inversely correlated with GA (r = − 0.328, *P* = 0.036) and FBW (r = − 0.324, *P* = 0.039). miR-363 recorded negative correlations with BMI (r = − 0.315, *P* = 0.045) and abnormal Doppler (r = − 0.26, *P* = 0.04) and a positive correlation with GA (r = 0.412, *P* = 0.006). MALAT-1 was negatively correlated with maternal age (r = − 0.341, *P* = 0.029) and albuminuria (r = − 0.35, *P* = 0.025), while showed a positive correlation with GA (r = 0.322, *P* = 0.04). HOTAIR was positively correlated with albuminuria (r = 0.274, *P* = 0.039). This correlations link the deregulated levels of our studied ncRNAs with PE pathology.

### Performance of investigated serum ncRNAs in PE diagnosis, onset and severity

**Figure 2:**
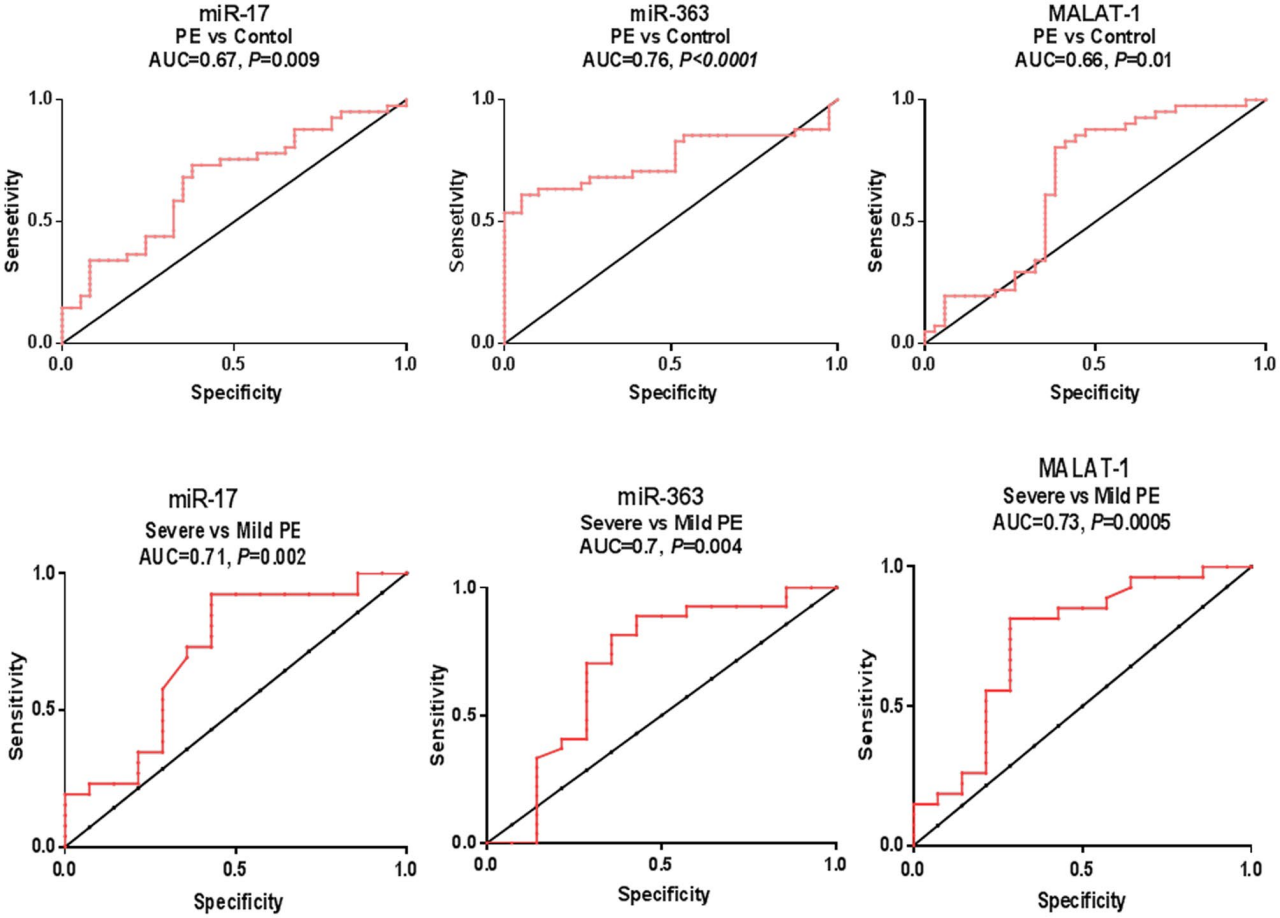
Diagnostic performance of serum miR-17, miR-363 and MALAT-1 and their association with severity in women with PE. ROC curve analysis of studied parameters to discriminate PE group (n = 82) versus healthy pregnancies (n = 78) and severe (n = 54) versus mild PE (n = 28).

Serum miR-17, miR-363, and MALAT-1 distinguished PE patients from the healthy controls in the ROC curve analysis with AUC = 0.67, *P* = 0.009, sensitivity (SN) = 73% and specificity (SP) = 62% at a cut off  > 1.6 (fold, 2^−∆∆Ct^) for miR-17; AUC = 0.76, *P* < 0.0001, SN = 61% and SP = 95% at a cut off < 0.17-fold for miR-363, and AUC = 0.66, *P* = 0.01, SN = 80% and SP = 62% at a cut off < 0.62-fold for MALAT-1 (Fig. 2). By comparison, miR-363 was a potential discriminator and seems to have better diagnostic accuracy than miR-17 and MALAT-1, but differences between AUCs didn't reach statistical significance (difference = 0.09, *P* = 0.079, difference = 0.1, *P* = 0.11, respectively).

miR-17, miR-363 and MALAT-1 were potential discriminators of severe from mild PE cases with AUC = 0.71, *P* = 0.002, SN = 92.31% and SP = 57.14% at a cut off > 1.47-fold for miR-17; AUC = 0.7, *P* = 0.004, SN = 70.37% and SP = 71.43% at a cut off < 0.33-fold for miR-363, and AUC = 0.73, *P* = 0.0005, SN = 81.48% and SP = 71.43% at a cut off < 0.66-fold for MALAT-1 (Fig. 2).

**Figure 3:**
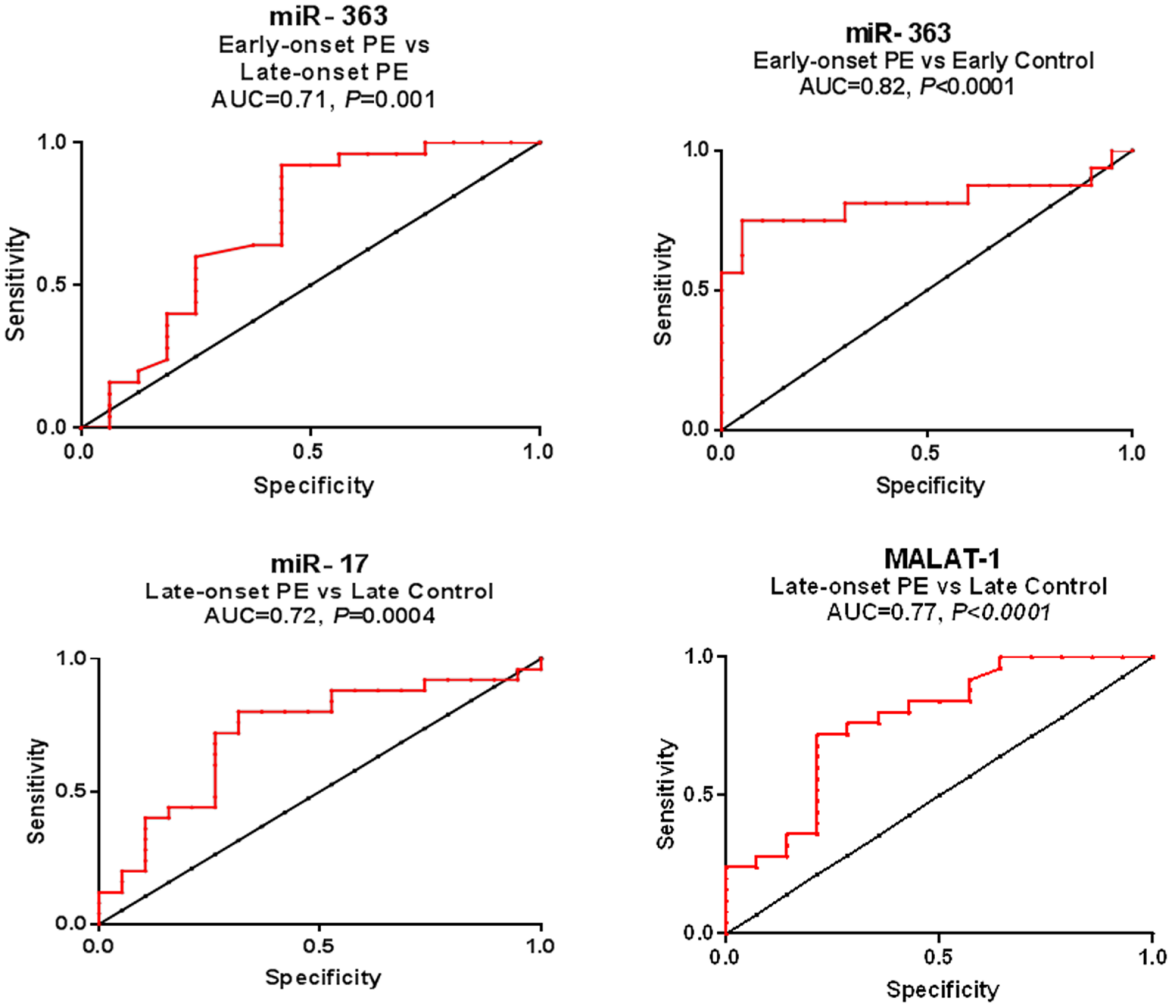
Diagnosis of either early- or late-onset PE using serum miR-363, miR-17 and MALAT-1. ROC curve analysis of studied parameters to discriminate early-onset PE (n = 32) versus late-onset (n = 50), early-onset PE (n = 32) vs early controls (n = 40), and late-onset PE (n = 50) vs late controls (n = 38).

Only miR-363 was a potential discriminator of early-onset from late-onset PE cases (AUC = 0.71, *P* = 0.001) with SN = 92% and SP = 56.25% at a cut off < 0.018-fold. Moreover, it was the only studied ncRNA that distinguished early-onset PE patients from early controls (AUC = 0.82, *P* < 0.0001) with SN = 75% and SP = 95% at a cut off < 0.14-fold. On the other hand, miR-17 (AUC = 0.72, *P* = 0.0004) and MALAT-1 (AUC = 0.77, *P* < 0.0001) were potential discriminators of late-onset PE patients from late controls with SN = 80% and SP = 68% at a cut off > 3.1-fold for miR-17, and SN = 72% and SP = 79% at a cut off < 0.52-fold for MALAT-1 (Fig. 3). All cutoff values were expressed as fold change (2^−∆∆Ct^) and determined as the value that maximized the sum of SN and SP.

### Association of studied parameters with early PE and PE severity using logistic regression analysis

The variables associated with early PE (early-onset PE vs early controls) (Table 4) and its severity (severe vs mild PE) (Table 5) were identified using univariate and multivariate logistic regression analyses. miR-363 along with clinical parameters; SBP, DBP, MAP, CRP, creatinine, IUGR, abnormal Doppler and low amniotic fluid come out to be associated with early PE (*P* < 0.05) in the univariate analysis. miR-363 together with SBP come out as the final independent variables associated with early PE in the multivariate analysis with adjustment by maternal age and GA as cofounders. Additionally, MALAT-1 along with clinical data; SBP, DBP, MAP, creatinine, uric acid, IUGR and abnormal Doppler were shown to associate with PE severity in the univariate analysis for (*P* < 0.05). Then, MALAT-1 together with SBP and serum uric acid turned out as the final independent variables associated with the severity of PE in the multivariate analysis with adjustment by maternal age.

**Table 4:** Association of biomarkers, maternal and fetal characteristics with early-onset PE compared with early controls using logistic regression analysis.

Parameter	Beta coefficient	SE	*P * value	OR	95% CI
**Univariate analysis**					
miR-363	− 1.63	0.65	0.012*	0.195	0.05–0.69
SBP	0.47	0.16	0.004*	1.6	1.16–2.19
DBP	0.71	0.35	0.044*	2.03	1.02–3.96
MAP	0.4	0.14	0.0035*	1.48	1.14–1.93
CRP	0.042	0.013	0.0008*	1.04	1.02–1.07
Creatinine	3.08	1.03	0.0028*	21.74	2.89–63.63
IUGR	2.44	0.677	0.0007*	7	2.06–19.83
Abnormal Doppler	2.14	0.68	0.0002*	9	2.74–26.98
Low amniotic fluid	2.73	0.68	> 0.0001*	63	13.7–239.4
**Multivariate analysis**^**a**^					
miR-363	− 1.3	0.7	0.02*	0.21	0.04–0.7
SBP	0.495	0.2	0.005*	1.75	1.6–2.1
Constant	− 9.57				

Univariate analysis was done using early-onset PE cases, n = 32 cases and early healthy pregnancies, n = 40 matched with gestational weeks at delivery (*P* > 0.05). Significant variables were then entered into stepwise forward multivariate analysis (*P* < 0.05 for entering and *P* < 0.1 for removal from the model). −2 log likelihood of the model*, P* < 0.0001. *Statistically significant, *P* < 0.05. CI, confidence interval; CRP, C-reactive protein; DBP, diastolic blood pressure; FBW, fetal birth weight; IUGR: intrauterine growth restriction; MAP, mean arterial pressure; OR, odds ratio; SBP, systolic blood pressure;  SE, standard error.

Bold values indicate statistical significance.

^a^Controlled by maternal age and gestational age as covariates, OR (95% CI) = 0.85 (0.350–2.01) and 0.66 (0.28–1.71), respectively.

**Table 5:** Association of biomarkers, maternal and fetal characteristics with severe PE compared with mild PE using logistic regression analysis.

Parameter	Beta coefficient	SE	*P * value	OR	95% CI
**Univariate analysis**					
miR-17	0.045	0.04	0.27	1.05	0.96–1.13
miR-363	2.8	3.01	0.35	16.5	0.04–60.59
MALAT-1	− 2.69	1.16	0.02*	0.068	0.007–0.66
GA	− 0.093	0.17	0.59	0.91	0.654–1.271
SBP	0.1	0.03	0.002*	1.11	1.039–1.18
DBP	0.29	0.093	0.002*	1.34	1.11–1.601
MAP	0.2	0.062	0.0015*	1.22	1.08–1.38
CRP	0.003	0.009	0.77	1.002	0.986–1.02
Creatinine	3.77	1.65	0.023*	43.42	1.69–110.7
Uric acid	2	0.866	0.021*	7.37	1.349–40.23
IUGR	2.48	0.87	0.0041*	12	2.198–65.51
Abnormal Doppler	2.94	1.11	0.008*	18.9	2.15–66.27
Low amniotic fluid	0.82	0.67	0.223	2.67	0.608–8.447
**Multivariate analysis**^**a**^					
MALAT-1	− 2.49	1.13	0.027*	0.068	0.008–0.68
SBP	0.169	0.07	0.015*	1.12	1.04–1.16
Uric acid	2.02	1.039	0.049*	6.83	1.001–23.5
Constant	– 33.17				

Univariate analysis was done using mild PE, n = 28; severe PE, n = 54 cases. Significant variables were then entered into stepwise forward multivariate analysis (*P* < 0.05 for entering and *P* < 0.1 for removal from the model). −2 log likelihood of the model*, P* < 0.0001. *Statistically significant, *P* < 0.05. CI, confidence interval; CRP, C-reactive protein; DBP, diastolic blood pressure; GA, gestational age; IUGR: intrauterine growth restriction; MAP, mean arterial pressure; SBP, systolic blood pressure; SE, standard error.

Bold values indicate statistical significance.

^a^Controlled by maternal age as covariate, OR (95%CI) = 0.8 (0.33–1.9).

### Results of functional analysis

**Figure 4:**
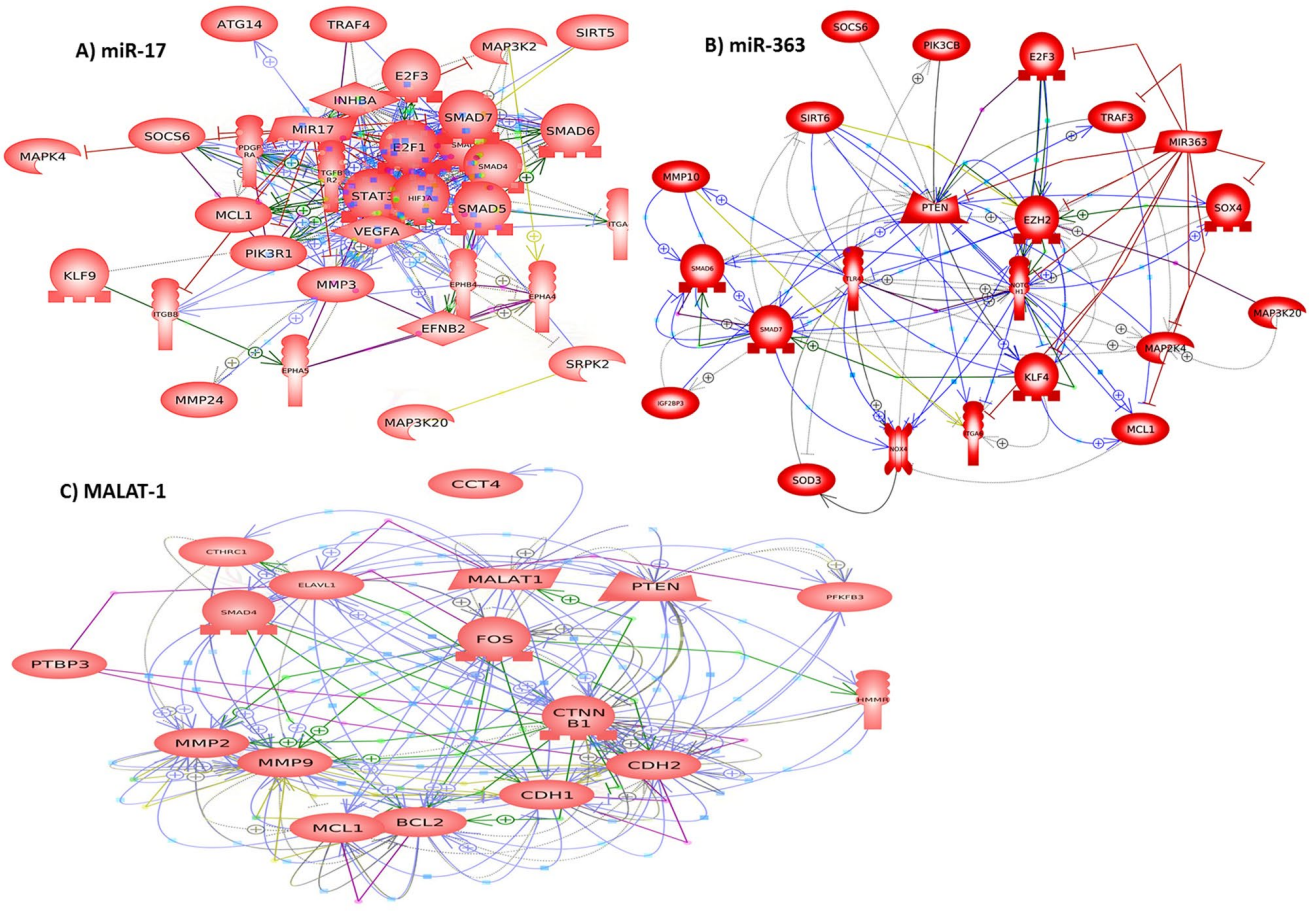
Construction of non-coding RNA-related PPI networks linked to PE. (**A**) miR-17, (**B**) miR-363 and (**C**) MALAT-1. Pathway Studio online software was used. Data relevant to KEGG software http://www.kegg.jp/kegg/kegg1.html.

We listed the selected target genes of miR-17, miR-363 and MALAT-1 most functionally linked to PE, their protein–protein interaction (PPI) *P* value, gene ontology (GO) biological process and KEGG pathways of the PPI in Table 6. The PPI network construction for miR-17, miR-363 and MALAT-1 is visualized in Fig. 4.

**Table 6:** Bioinformatics analysis of the selected placental-related non-coding RNAs-related genes and protein–protein interactions linked to PE.

Non-coding RNA	Target genes	PPI *P* value	Gene ontology for PPI network		KEGG pathway analysis for PPI network	
			Biological process	Strength (FDR)	Pathway	Strength (FDR)
miR-17	VEGFA, EPHB4, EFNB2, EPHA4, EPHA5, TGFBR2, HIF1A, ITGA4, ITGB8, STAT3, E2F1, MAP3K2, MAPK4, SMAD1, SMAD4, SMAD5, SMAD6, SMAD7, PIK3R1, PDGFRA, MCL-1, MMP24, MMP3, SRPK2, KLF9, MAP3K20, TRAF4, E2F3, SIRT5, SOCS6, ATG14, INHBA	1.00E-16	Regulation of vascular endothelial cell proliferation	1.72 (0.00026)	TGF-beta signaling pathway	1.53 (7.06e−11)
			Developmental cell growth	1.05 (0.0099)	VEGF signaling pathway	1.43 (6.33e−06)
			Angiogenesis	1.2 (1.65e−12)	HIF-1 signaling pathway	1.51 (1.23e−11)
			Enzyme linked receptor protein signaling pathway	1.12 (6.78e−22)	MAPK signaling pathway	1.24 (3.53e−14)
			Protein stabilization	0.88 (0.0078)	JAK-STAT signaling pathway	1.2 (2.29e−07)
			Protein phosphorylation	0.95 (3.24e−16)	PI3K-Akt signaling pathway	1.24 (8.81e−17)
			Cellular response to oxidative stress	0.85 (0.0033)	Regulation of insulin receptor signaling pathway	1.08 (0.0356)
			Extracellular matrix organization	0.73 (0.0095)	SMAD protein complex assembly	1.96 (0.0017)
			Regulation of protein kinase activity	0.83 (6.30e−09)	Growth hormone receptor signaling pathway	1.57 (0.0065)
			Embryo development	0.8 (4.77e−09)	Ephrin receptor signaling pathway	1.6 (7.36e−12)
			Regulation of DNA-binding transcription factor activity	0.67 (0.0071)	Epidermal growth factor receptor signaling pathway	1.08 (0.0356)
			Apoptosis	1.07 (0.00021)	Integrin-mediated signaling pathway	1.39 (0.0159)
			Platelet activation	0.9 (0.0217)		
			Tissue remodeling	1.13 (0.0013)		
miR-363	KLF4, ITGA6, PTEN, EZH2, NOX4, SOD3, SOX4, MAP2K4, MAP3K20, MCL1, E2F3, SMAD6, SMAD7, TRAF3, TLR4, IGF2BP3, TGFIF1, NOTCH1, MMP10, PIK3CB, SIRT6, SOCS6	4.03E−11	Tissue remodeling	1.18 (0.0054)	Notch signaling pathway	1.78 (4.79e−08)
			Regulation of DNA-binding transcription factor activity	0.98 (3.00e−05)	Canonical Wnt signaling pathway	1.19 (0.0052)
			Regulation of developmental growth	0.98 (0.00037)	TGF-beta signaling pathway	1.24 (0.0026)
			Cellular response to reactive oxygen species	1.06 (0.0096)	VEGF signaling pathway	1.21 (0.0142)
			Regulation of angiogenesis	0.84 (0.0107)	HIF-1 signaling pathway	1.16 (0.0037)
			Regulation of signal transduction	0.61 (4.69e−10)	JAK-STAT signaling pathway	1.08 (0.0016)
			Regulation of cellular response to stress	0.79 (0.00036)	PI3K-Akt signaling pathway	0.92 (0.00053)
			Platelet activation	1.08 (0.0090)	Activation of MAPK activity	1.2 (0.00019)
			Apoptosis	1.03 (0.0072)	Insulin signaling pathway	0.85 (0.0455)
			Regulation of protein kinase signaling	1.07 (0.00059)	Estrogen signaling pathway	0.86 (0.0454)
			Protein stabilization	0.94 (0.0176)		
			Regulation of protein phosphorylation	0.77 (3.55e−08)		
			Enzyme linked receptor protein signaling pathway	0.61 (0.0121)		
			Tissue regeneration	1.25 (0.0201)		
MALAT-1	PTEN, SMAD4, MMP2, MMP9, E-cadherin, N-cadherin, β-catenin, Elavl1, CTHRC1, CCT4, HMMR, MCL-1, Bcl-2, PTBP3, PFKFB3, C-FOS	5.09E−12	Extracellular matrix organization	1.02 (0.0057)	Platelet-derived growth factor receptor signaling pathway	1.66 (0.0083)
			Embryo implantation	1.6 (0.0097)	SMAD protein signal transduction	1.43 (0.0170)
			Protein stabilization	1.58 (2.53e−09)	Ephrin receptor signaling pathway	1.3 (0.0252)
			Apoptosis	1.24 (0.0042)	Regulation of epidermal growth factor receptor signaling pathway	1.29 (0.0256)
			Cellular response to oxidative stress	1.15 (0.0023)	PI3K-Akt signaling pathway	0.83 (0.0359)
			Enzyme linked receptor protein signaling pathway	0.83 (0.0026)	HIF-1 signaling pathway	1.2 (0.0310)
			Developmental growth	0.84 (0.0402)	Estrogen signaling pathway	1.37 (0.00056)
			Intracellular signal transduction	0.69 (0.0090)	Regulation of canonical Wnt signaling pathway	1.1 (0.0462)
			Cell migration	0.68 (0.0195)	TGF-beta signaling pathway	1.25 (0.0277)
			Embryo development	0.64 (0.0256)	Response to growth factor	0.79 (0.0211)

The PPI and functional enrichment analysis for the PPI were conducted using SPRING software. Data relevant to KEGG pathways http://www.kegg.jp/kegg/kegg1.html. PPI, protein–protein interactions. FDR, false discovery rate.

## Discussion

New molecular biomarkers that give mechanistic insights in PE and could boost its screening, early detection and prognosis are urgently needed to reduce PE-associated maternal death. In this exploratory study, we have conducted a systematic bioinformatics analysis and selected 4 PE-related ncRNAs for our biomarker study. Results showed that serum miR-17, miR-363 and MALAT-1 expression profiles were surrogate biomarkers of PE risk and severity. Furthermore, we are the first to show an association between serum miR-363 and early-onset PE, and between serum MALAT-1 downregulation and PE progression.

In PE patients, we observed serum MALAT-1 downregulation with miR-17 upregulation, which verify their cross-interaction in PE development and progression. To explain, miR-17, MALAT-1 and also HOTAIR commonly regulate vascular endothelial growth factor A (VEGFA)^15,16^, through which they could presumably crosstalk to regulate angiogenesis. Taking into account that MALAT-1 and HOTAIR were shown to act as ceRNAs for miR-17^17,18^, thus MALAT-1 underexpression could elevate miR-17 with concomitant downregulation of miR-17 target genes, including VEGFA resulting in impaired placental angiogenesis. On the other hand, both MALAT-1 and HOTAIR are estrogen receptor α (ERα) and hypoxia inducible factor-1α (HIF1A) transcriptional targets^19–21^. As HIF1A is a predicted target for miR-17 (Table 6) and ERα is a known target of miR-17-92 family^12^, one may speculate that miR-17 upregulation could possibly affect HIF1A and ERα expression and hence the transcription of MALAT-1 gene. However, this relation should be further investigated.

Although several target genes and biological pathways have been previously identified for our selected ncRNAs and were linked to PE pathogenesis, we have conducted target gene analysis and PPI network analysis to further explain the role of miR-17, MALAT-1 and miR-363 in PE pathogenesis (Table 6 and Fig. 4). We found several common targets and biological pathways for miR-17, miR-363 and MALAT-1 which are involved in VEGF signaling pathway, HIF signaling, ephrin receptor signaling, transforming growth factor (TGF)-β/SMAD signaling as well as epidermal growth factor receptor (EGFR), MAPK, PI3K/Akt, JAK/STAT and canonical Wnt signaling pathways. Notably, these pathways are known to regulate many trophoblastic cell activities^4,5^. Together, these data clarify that these miRNAs could interplay in PE development and progression.

The observed elevation of serum miR-17-5p in PE coincides with previously reported in whole maternal peripheral blood^22^ and placenta^9,11,23^ among PE patients. Indeed, circulating miRNAs in pregnant women mainly originate from the placenta. Conversely, miR-17 was not altered in plasma^9^, whole maternal blood or placenta of PE patients^24,25^. Mechanistically, miR-17 targets a set of genes including, VEGFA, ephrin type-B receptor 4 (EPHB4), EPHA4, EPHA5, ephrin-B2 (EFNB2), and matrix metalloproteinases (Table 6, Fig. 4). These genes are cardinal for vascular remodeling and CTB invasion during placentation^11^. In early placental development low levels of miR-17 maintains trophoblastic differentiation by upregulating EFNB2 and EPHB4, but in PE miR-17 overexpression suppresses these genes causing inhibition of trophoblastic invasion and defective placental vasculature^11,23^. Moreover, miR-17 promotes oxidative stress-induced apoptosis via targeting STAT3 which interfere with PE development^26^.

We observed elevated serum miR-17 in severe PE as well as in late-onset PE patients vs late controls, but not among early and late gestational stages of PE. Similarly, miR-17 was upregulated in severe PE placentas^9^ and in whole maternal peripheral blood in severe PE^22^. Conversely, higher postpartum expression of miR-17-5p was observed within late PE patients compared to those with early PE^22^. However, placental, plasma and whole maternal blood miR-17 were not correlated with either PE severity or onset in other studies^9,24,25^. Our target gene analysis could justify the link between miR-17 and PE severity on the basis that miR-17 is predicted to target TGFBR2, SMAD6/SMAD7 and SMAD4/SMAD5 thus could modulate TGF-β signaling. Indeed, this pathway was shown to be central to PE pathogenesis^27^.

The observed association of miR-17 with BMI is consistent with its adipogenesis-promoting actions^28^, confirming that obesity is a risk factor for PE, with many common mechanisms interplay to link obesity with a higher risk of PE^29^. The negative correlation between miR-17 and GA is consistent with that miR-17 expression was altered in porcine placenta of different GAs, proposing that miR-17 declines as GA advances^30^. miR-17 downregulation was also reported in third trimester placentas compared with first trimester ones^31^. This correlation is attributed to that miR-17 targets VEGFA which is responsible for increasing the growth of placental vasculature as GA increases^32^. Although previous reports were discrepant^33,34^, the negative correlation between miR-17 and FBW intensifies that miR-17 can be used as a prognostic biomarker for maternal diseases affecting birth weight^35^. Despite controversial reports^24,25^, our results might link dysregulated miR-17 and IUGR, a well-known risk factor of PE especially the severe form.

The observed serum miR-363 downregulation mimics prior reports of placental miR-363 underexpression in PE^9,36,37^. Conversely, plasma and serum miR-363 expression did not alter in PE throughout the 3 trimesters^9,38^. miR-363 is an anti-apoptotic miRNA; its downregulation leads to excessive placental trophoblast apoptosis exhibited in PE^38^. Furthermore, inhibition of trophoblast cell differentiation and invasion was associated with decline of placental miR-363 expression^39^. To explain, we showed that miR-363 is predicted to target several genes, among them integrin-A6 (ITGA6), a receptor for cell-extracellular matrix interaction important for trophobalst migration and invasion; and Kruppel-like factor 4 (KLF4), a transcription factor which regulates angiogenesis.

The observed decline of serum miR-363 in severe and early-onset PE coincided with previously reported in PE placenta^9,36,37^. However, plasma miR-363 was not correlated with PE severity^9^ or onset^38^ in other studies. miR-363 deregulation associates with PE severity by targeting placental sodium coupled neutral amino acid transporters causing variation in amino acids transport and nutrient transfer, ultimately leading to PE pathology^39^. Moreover, aberrantly expressed miR-363 together with EZH2 and nudix hydrolase 21 were shown to influence CTBs growth and migration^40^. The observed miR-363 downregulation in both early-onset and severe PE supports the notion that early-onset PE is often more severe than late-onset PE and largely originates from poor placentation in the first trimester^41^. Both PE phenotypes exhibit intensified systemic inflammatory responses exposing vessels and cells to overwhelming oxidative stress, with consequent apoptosis^37^.

The recorded negative correlation between miR-363 and BMI is consistent with its anti-adipogenic properties^42^. The positive correlation between serum miR-363 and GA coincides with placental miR-363 upregulation in third versus first trimester^31^. However, serum miR-363 was not correlated with GA throughout the three trimesters in PE^37^.

The observed downregulation of serum MALAT-1 in PE matches the previously reported data in placenta, umbilical cords and mesenchymal stem cells (MSCs) derived from PE patients^13,15,43^. Using a bioinformatics approach, we identified a MALAT-1-related PPI network linked to PE, including PTEN, SMAD4, MMP2, MMP9, E-cadherin, N-cadherin, β-catenin, Elavl1, CTHRC1, CCT4, HMMR, MCL-1, BCl-2, PTBP3, PFKFB3 and C-FOS. This PPI was enriched in extracellular matrix organization, cell migration, embryo development, cellular response to oxidative stress and apoptosis and is involved in platelet-derived growth factor receptor signaling, TGF-β/SMAD signaling, ephrin receptor signaling, EGFR signaling, HIF signaling and estrogen signaling as well as PI3K/Akt and Wnt/β-catenin signaling pathways. To put this in context, placental MALAT-1 is important for trophoblast invasion; its silencing in placental trophoblastic cells suppressed invasion, migration and motility through influencing CTHRC1 and CCT4 expression^13,44,45^. Interestingly, MALAT-1 is required for capillary formation and angiogenesis^46–48^; its knockdown markedly reduced angiogenesis by regulating VEGF, TGF-β, fibroblast growth factor-2 and tumour necrosis factor-α expressions^19,46,48^. Moreover, MALAT-1 modulates cell proliferation by acting as a ceRNA via interactions with miRNAs during the recurrent abortion occurrence^47^.

We further showed a decline in serum MALAT-1 in severe PE as well as in late-onset PE patients vs late controls, but not among early- and late-onset PE cases. Similarly, MALAT-1 underexpression was involved in severe PE within umbilical cords and MSCs from PE patients through affecting MSCs proliferation, angiogenesis, apoptosis, migration, invasion and immunosuppressive properties^15^. Conversely, MALAT-1 was not correlated with the onset of PE in placenta, umbilical cord and MSCs from PE patients^13,15^.

Interestingly, we found negative associations of MALAT-1 with maternal age and albuminuria which were in contrast with previous findings^13,49,50^. In fact, PE is more common in women who become pregnant at advanced maternal age^51^. Therefore, the decline in MALAT-1 with advanced maternal age presumably contributes to PE. Moreover, the inverse correlation between MALAT-1 and albuminuria could exacerbate PE pathology presumably through a dysregulated β-catenin/MALAT-1 axis which promotes podocyte malfunction, albuminuria and finally kidney fibrosis^52^. Although the correlation between MALAT-1 and GA was discrepant^49,50^, we found a positive correlation.

We reported a lack of association between serum HOTAIR and PE risk. Conversely, HOTAIR was upregulated in PE placenta, where it reduced cell proliferation and enhanced apoptosis by increasing caspase-3^14^. HOTAIR was also elevated in severe PE, and altered HOTAIR was implicated in PE development^53^. This matches with our observed positive correlation between HOTAIR and albuminuria.

There are several limitations in our study. First, this was a case–control study in which the included patients and controls were recruited only from one hospital. Second, a modest sample size was used; however, cases were extremely filtered due to our rigorous inclusion and exclusion criteria. Third, no samples were collected before the onset of PE, and the lack of longitudinal follow up may limit the certainty of prognosis of PE from mild to severe. Finally, larger-scale predictive studies are warranted to replicate our results. Nevertheless, our data provide new clinical tools that might be implicated in genomic analysis in individualized testing with the wide availability and the technical ease of ncRNAs measurement. Future work would include establishing a normal range of the expression levels rather than using fold change to facilitate clinical application.

## Conclusion

Our study accentuates miR-17, miR-363 and MALAT-1 as potential new biomarkers for PE and its severity. miR-363 was associated with early PE and MALAT-1 was associated with severe PE. By functional analysis, miR-17, miR-363 and MALAT-1 could interplay in PE pathogenesis through common targets and signaling pathways. Our data appraise the progresses in finding new biomarkers for diagnosing PE and evaluating severity and onset of PE, and also highlights areas for future research.

## Subjects and methods

### Patients

This prospective study involved 160 Egyptian pregnant females who received routine obstetric examination at the Department of Obstetrics and Gynecology, Kasr Al-Ainy hospital, Cairo University. The study included eligible 82 pregnant females diagnosed with PE and 78 healthy normotensive age-matched pregnant women without proteinuria or any complications as the control group.

Full history taking, physical and clinical examination were done for all participants. The medical records of each participant were revised and all relevant data were used in the study, including information on risk factors, pregnancy history and perinatal outcome. PE was diagnosed as new-onset of hypertension (SBP ≥ 140 mmHg and/or DBP ≥ 90 mmHg on at least 2 occasions 4 h apart after the 20th gestational week combined with new-onset proteinuria according to the guidelines of the American College of Obstetricians and Gynecologists (ACOG 2013)^54^. Significant protein excretion was defined as ≥ 300 mg protein/24 h-urine sample or ≥ 1+ urine dipstick testing of two random urine samples collected at least 4 h apart. Blood samples were collected after diagnosis of PE for preeclamptic women, and from normal pregnant women at admission to the maternity ward at Kasr Al-Ainy hospital between October 2017 and March 2018. Blood pressure (BP) was measured and PE was diagnosed before blood sampling. Patients with PE were subdivided based on severity of clinical symptoms to mild and severe cases. Severe PE was defined as SBP ≥ 160 mmHg and/or DBP ≥ 110 mmHg on 2 different occasions, accompanied with proteinuria (≥ 2 g/24 h or ≥ 2+ dipstick) and the presence of persistent headache, epigastric right upper quadrant abdominal pain, vomiting, elevation of uric acid, increased serum creatinine and liver enzymes, thrombocytopenia, red cell breakdown, visual impairment, swelling, shortness of breath due to pulmonary edema and IUGR^54^. Mild PE was defined as SBP ≥ 140 to 159 or DBP ≥ 90 to 109 mmHg in 2 different occasions, accompanied with proteinuria (≥ 300 mg to 1.99 g/24 h or ≥ 1+ dipstick)^54^. Early-onset PE was defined as developing clinical manifestations of PE at ≤ 34 weeks of gestation whereas cases regarded as late-onset PE if occurred > 34 gestational weeks^10^.

BP was measured at admission for controls and PE patients. We expressed gestational weeks as gestational age (GA) to define the pregnancy weeks using ultrasound imaging for fetal measurements. GA was measured and recorded for every patient at admission and before blood sampling. IUGR was defined as weight below the 10th percentile for the GA as measured by an ultrasound device. BMI was defined as the body mass divided by the square of the body height, and was expressed in units of kg/m^2^. BMI was determined using the mother’s body weight at the time of inclusion. Blood samples were collected between 20 and 40 weeks of gestation.

Inclusion criteria were pregnant females from 20 to 40 gestational weeks and none of these subjects had any invasive procedure. Women with pre-existing hypertension, hemostatic abnormalities, cancer, twin pregnancy, intrauterine fetal death, gestational diabetes, and cardiovascular, autoimmune, renal and hepatic diseases were excluded.

All participants signed an informed consent and all experiments were approved by the ethical committee of the Faculty of Pharmacy, Cairo University, Cairo, Egypt (BC2074). All methods were carried out in accordance with guidelines and regulations in Helsinki declaration.

### Sample collection for RNA assay

About 5 mL maternal venous blood was drawn from each participant by vein puncture and collected in a plain tube. Blood was left to clot at room temperature for 30 min and then centrifuged at 2000*g* for 15 min. Sediment- and hemolysis-free supernatants were quickly removed and aliquoted. Aliquots were immediately frozen at – 80 °C until RNA extraction. For analysis, serum samples were thawed once on ice and centrifuged at 3000*g* for 5 min to avoid the presence of any traces of red blood cells and other cellular debris which could affect the miRNAs and lncRNAs profile.

### RNA extraction

Total RNA was isolated from 200 µL serum using the miRNeasy Serum/Plasma kit (Qiagen, Germany) following the manufacturer's instructions. Total RNA concentration and purity were analyzed using Bioanalyzer Agilent RNA 6000 picoassay. RNA was used for detection of miRNAs and lncRNAs.

### miRNAs assay using RT-qPCR

Briefly, 0.1 μg of total RNA was reverse transcribed using the miScript II RT kit (Qiagen) in a total 20 µL reaction volume according to the manufacturer's instructions. The thermal parameters were 60 min at 37 °C and 5 min at 95 °C. Quantitative real-time PCR was then performed in a total 20 µL reaction volume on Rotorgene Q system (Qiagen) using the miScript SYBR Green PCR kit (Qiagen) and the provided miScript Universal Primer (reverse primer) and specific primers (forward primers) for hsa-miR-17-5p, hsa-miR-363-3p as well as SNORD68 as the internal control according to the manufacturer’s instructions. Briefly, real-time PCR was conducted in 20 µL reaction mixtures where 2.5 μL of appropriately diluted cDNA template was mixed with 5.5 μL RNase free water, 10 μL miScript SYBR Green PCR Master Mix and 2 μL miScript forward and reverse primers. The PCR thermal conditions were 15 min at 95 °C, 40 cycles of 15 s at 94 °C followed by 30 s at 55 °C and 30 s at 70 °C.

### lncRNAs assay using RT-qPCR

Reverse transcription (RT) was conducted on 0.1 μg of total RNA in a 20 µL RT reaction with the high capacity cDNA Reverse Transcriptase kit (Applied Biosystems, USA) following the manufacturer’s instructions. The thermal cycler conditions were as follows: 10 min at 25 °C, 110 min at 37 °C, and 5 s at 95 °C. Expression levels of HOTAIR and MALAT-1 were evaluated by qPCR using GAPDH as the housekeeping gene. Customized primers and the Maxima SYBR Green PCR kit (ThermoFischer, USA) were used to prepare the PCR master mixture following the manufacturer’s protocol. The primer sequences were HOTAIR-forward 5′-GGTAGAAAAAGCAACCACGAAGC-3′, HOTAIR-reverse 5′-ACATAAACCTCTGTCTGTGAGTGCC-3′, MALAT-1-forward 5′-AAAGCAAGGT-CTCCCCACAAG-3′, MALAT-1-reverse 5′-GGTCTGTGCTAGATCAAAAGGCA-3′, GAPDH-forward 5′-GAAGGTCGGAGTCAACGGATT-3′, and GAPDH-reverse 5′-CGCTCCTGGAAGATGGTGAT-3′. Primer specificity was checked using the NCBI Primer-BLAST tool (https://www.ncbi.nlm.nih.gov/tools/primer-blast/). Real-time PCR was performed on Rotorgene Q system (Qiagen) in 20 µL reaction mixtures with the following conditions: 95 °C for 10 min, followed by 40 cycles at 95 °C for 15 s and 60 °C for 60 s.

Expression analysis of miRNAs and lncRNAs relative to internal control were done using 2^−ΔCt^ method, where ΔCt = Ct_gene_ − Ct_internal control_. Fold change was calculated with the formula 2^−∆∆Ct^, where ∆∆Ct = ΔCt_patient_ − ΔCt_control group_.

### Selection of lncRNAs and miRNAs using bioinformatics analysis

#### Selection of PE-associated lncRNAs

We used the lncRNA disease database (http://www.cuilab.cn/lncrnadisease) to screen for PE-associated lncRNAs. Seven candidate lncRNAs were revealed (H19, MALAT-1, HOTAIR, LOC391533, LOC284100, CEACAMP8 and SPRY4-IT1) (Supplementary Table S1). Using PubMed search (http://www.ncbi.nlm.nih.gov/pubmed/), MALAT-1 and HOTAIR were selected for this study based on their biological relevance to PE^13,14^.

#### Selection of PE-associated miRNAs

PE-associated miRNAs were screened using the Human microRNA Disease Database (HMDD) v3.2 (https://www.cuilab.cn/hmdd). More than 95 different miRNAs were recorded in relation with PE. Members of miR-17-92 family, namely miR-17, miR-18a and miR-20a and members of its paralog 106a-363 cluster, namely miR-106a, miR-18b, miR-20b and miR-363 were PE candidates (Supplementary Table S2).

### miRNA-lncRNA interaction analysis

The starBase platform (http://starbase.sysu.edu.cn/) was used to check the interaction between candidate miRNAs and the lncRNAs; MALAT-1 and HOTAIR. Some of the results are shown in supplementary Table S3. The database reported interactions of HOTAIR with miR-17-5p, miR-20a-5p, miR-20b-5p and miR-106a-5p as well as interactions of MALAT-1 with miR-17-5p, miR-20a-5p, miR-363-3p, miR-20b-5p and miR-106a-5p. To filter these data, we searched the PubMed for experimentally validated lncRNA-miRNAs interactions. We found previously reported direct interactions between MALAT-1 and miR-17-5p^17^, MALAT-1 and miR-363-3p^55^, and HOTAIR and miR-17-5p^18^ based on a reporter assay. Thus, MALAT-1, HOTAIR, miR-17-5p and miR-363-3p were selected for this study.

### Target gene analysis and construction of protein–protein interaction networks

For miRNAs, the online databases, TargetScan (http://www.targetscan.org/vert_72/) and miRDB (http://mirdb.org/), were used to find the predicted target genes for the miR-17 and miR-363. For lncRNAs, the starBase platform (http://starbase.sysu.edu.cn/) was used to screen the candidate lncRNA-RNA interactions. We filtered the output by selecting protein-coding genes.

Target genes were then analyzed using CapitalBio Molecule Annotation System 3.0 software to determine the biological roles of the lncRNA and miRNA-target protein-coding genes. Finally, the genes most related to PE pathogenesis in terms of biological process, molecular function and KEGG pathways were selected. The cutoff *P* value was 0.05.

We analyzed the relationships between the proteins (protein–protein interaction, PPI) encoded by the genes related to the selected ncRNAs (miR-17, miR-363 and MALAT-1) using STRING online software. We also used the STRING online software to conduct functional enrichments; GO and KEGG pathways to determine the involvement of each PPI in different biological pathways related to PE. The Pathway Studio Online Software was used to visualize the PPI network related to each selected placental-related ncRNA.

### Statistical analysis

Values are presented as mean ± SD, median (25%–75% percentiles), or number (percentage) when appropriate. Shapiro Wilk and Klomogrov Simirnov normality tests were used to check data normality. Data were compared using the parametric Student’s t test or the non-parametric Mann—Whitney U test when appropriate. The examined miRNAs, lncRNAs and CRP data were not normally distributed and their levels were compared by applying the Mann—Whitney U test. Fischer exact test was used to compare the categorical data. Receiver-operating-characteristic (ROC) analysis was carried out to evaluate the diagnostic and prognostic accuracy of molecular data. Area under the curve (AUC) < 0.6 was considered as non-significant, AUC ≥ 0.6 and < 0.7 as significant discriminator, AUC ≥ 0.7 and < 0.9 as potential discriminator, and AUC ≥ 0.9 as excellent discriminator. The associations between the biomarkers, early PE risk and PE severity were investigated using univariate followed by stepwise forward multivariate logistic regression analyses. Correlations between parameters were identified using Spearman correlation. Statistical significance was considered at *P* < 0.05. Statistical analyses were carried out using SPSS software v15 for Microsoft Windows (SPSS, Chicago, IL) and GraphPad Prism 7.0 (GraphPad Software, CA, USA).

### Ethics approval

(a) All experiments were approved by the ethical committee of the Faculty of Pharmacy, Cairo University, Cairo, Egypt (BC2074). (b) All methods were carried out in accordance with guidelines and regulations in Helsinki declaration.

## Supplementary Information

Supplementary Tables.

## Data Availability

The data that supports the findings of this study are available in the manuscript and in the supplementary material of this article.
