# Supplementary material for: Uncovering serum placental-related non-coding RNAs as possible biomarkers of preeclampsia risk, onset and severity revealed MALAT-1, miR-363 and miR-17
Source: Sci Rep. 2022 Jan 24;12:1249. doi: 10.1038/s41598-022-05119-9 (PMC8786922; doi:10.1038/s41598-022-05119-9)
Supplement: Supplementary file 1 — Supplementary Tables. [file 41598_2022_5119_MOESM1_ESM.docx]

**Supplementary Information**

**Uncovering serum placental-related non-coding RNAs as possible biomarkers of preeclampsia risk, onset and severity revealed MALAT-1, miR-363 and miR-17**

**Samy A. Abdelazim^a^*, Olfat G. Shaker^b^, Yehya Aly Hussien Aly^c^, Mahmoud A. Senousy^a^***

^a^Biochemistry department, Faculty of Pharmacy, Cairo University, Cairo, Egypt.

^b^Medical Biochemistry and Molecular Biology department, Faculty of Medicine, Cairo University, Cairo, Egypt.

^c^Pharmacist at Kasr Al-Ainy Hospital, Cairo University, Cairo, Egypt.

***Corresponding authors**

**Mahmoud A. Senousy (mohmoud.ali@pharma.cu.edu.eg)**

**Postal code: 11562**

**Mobile number: +201201712796**

**ORCID ID:** **https://orcid.org/0000-0003-3942-0275**

**Samy A. Abdelazim (samy.mohamed@pharma.cu.edu.eg)**

**Postal code: 11562**

**Mobile number: +201220777333**

**ORCID ID:** **https://orcid.org/0000-0003-1180-770X**

**Table S1 PE-related lncRNAs according to the lncRNA disease database**

| **LncRNA name** | **Disease name** | **Dysfunction type** | **Chr** | **Start** | **End** | **Strand** | **Species** | **Genbank** | | **Sequence** | **Reference** |
| --- | --- | --- | --- | --- | --- | --- | --- | --- | --- | --- | --- |
| H19 | pre-eclampsia | Mutation | chr11 | 1995176 | 2001466 | - | Human | [NR_131223](http://www.ncbi.nlm.nih.gov/nuccore/NR_131223) | [Gene](http://www.cuilab.cn/files/images/ldd/geneseq/H19.txt) / [RNA](http://www.cuilab.cn/files/images/ldd/rnaseq/H19.txt) | | [19570415](http://www.ncbi.nlm.nih.gov/pubmed/19570415) |
| **MALAT1** | **pre-eclampsia** | **Regulation** | **chr11** | **65497679** | **65504494** | **+** | **Human** | [**NR_002819**](http://www.ncbi.nlm.nih.gov/nuccore/NR_002819) | [**Gene**](http://www.cuilab.cn/files/images/ldd/geneseq/MALAT1.txt)**/**[**RNA**](http://www.cuilab.cn/files/images/ldd/rnaseq/MALAT1.txt) | | [**26722461**](http://www.ncbi.nlm.nih.gov/pubmed/26722461) |
| **HOTAIR** | **pre-eclampsia** | **Regulation** | **chr12** | **53962308** | **53974956** | **-** | **Human** | [**NR_003716**](http://www.ncbi.nlm.nih.gov/nuccore/NR_003716) | [**Gene**](http://www.cuilab.cn/files/images/ldd/geneseq/HOTAIR.txt)**/**[**RNA**](http://www.cuilab.cn/files/images/ldd/rnaseq/HOTAIR.txt) | | [**25807808**](http://www.ncbi.nlm.nih.gov/pubmed/25807808) |
| LOC391533 | pre-eclampsia | Regulation | N/A | N/A | N/A | N/A | Human | [N/A](http://www.ncbi.nlm.nih.gov/nuccore/N/A) | [Gene](http://www.cuilab.cn/files/images/ldd/geneseq/LOC391533.txt) / [RNA](http://www.cuilab.cn/files/images/ldd/rnaseq/LOC391533.txt) | | [24312300](http://www.ncbi.nlm.nih.gov/pubmed/24312300) |
| LOC284100 | pre-eclampsia | Regulation | N/A | N/A | N/A | N/A | Human | [N/A](http://www.ncbi.nlm.nih.gov/nuccore/N/A) | [Gene](http://www.cuilab.cn/files/images/ldd/geneseq/LOC284100.txt) / [RNA](http://www.cuilab.cn/files/images/ldd/rnaseq/LOC284100.txt) | | [24312300](http://www.ncbi.nlm.nih.gov/pubmed/24312300) |
| CEACAMP8 | pre-eclampsia | Regulation | chr19 | 43035869 | 43041248 | - | Human | [ENST00000413902](http://www.ncbi.nlm.nih.gov/nuccore/ENST00000413902) | [Gene](http://www.cuilab.cn/files/images/ldd/geneseq/CEACAMP8.txt) / [RNA](http://www.cuilab.cn/files/images/ldd/rnaseq/CEACAMP8.txt) | | [24312300](http://www.ncbi.nlm.nih.gov/pubmed/24312300) |
| SPRY4-IT1 | pre-eclampsia | Regulation | chr5 | 142317620 | 142318322 | - | Human | [NR_131221](http://www.ncbi.nlm.nih.gov/nuccore/NR_131221) | [Gene](http://www.cuilab.cn/files/images/ldd/geneseq/SPRY4-IT1.txt) / [RNA](http://www.cuilab.cn/files/images/ldd/rnaseq/SPRY4-IT1.txt) | | [24223182](http://www.ncbi.nlm.nih.gov/pubmed/24223182) |
| H19 | pre-eclampsia | Expression | chr11 | 1995176 | 2001466 | - | Human | [NR_131223](http://www.ncbi.nlm.nih.gov/nuccore/NR_131223) | [Gene](http://www.cuilab.cn/files/images/ldd/geneseq/H19.txt) / [RNA](http://www.cuilab.cn/files/images/ldd/rnaseq/H19.txt) | | [22832245](http://www.ncbi.nlm.nih.gov/pubmed/22832245) |

The search was done in the lncRNA disease database (<http://www.cuilab.cn/lncrnadisease>) by using disease "Pre-eclampsia". Selected lncRNAs are in bold.

**Table S2. PE-related miRNAs of the 17-92 and 106a/363 clusters revealed from HMDD v3.2**

| miRNA | Evidence code | Disease name | PMID |
| --- | --- | --- | --- |
| **hsa-mir-106a** | **tissue_expression_ns** | **Preeclampsia** | **25499681** |
| **hsa-mir-17** | **circulation_biomarker_prognosis_down** | **Preeclampsia** | **26339600** |
| **hsa-mir-17** | **target gene** | **Preeclampsia** | **22438230** |
| **hsa-mir-18a** | **circulation_biomarker_diagnosis_ns** | **Preeclampsia** | **25738738** |
| **hsa-mir-18b** | **circulation_biomarker_diagnosis_ns** | **Preeclampsia** | **25738738** |
| **hsa-mir-20a** | **target gene** | **Preeclampsia** | **22438230** |
| **hsa-mir-20a** | **tissue_expression_down** | **Preeclampsia** | **26992682** |
| **hsa-mir-20b** | **target gene** | **Preeclampsia** | **22438230** |
| **hsa-mir-363** | **tissue_expression_ns** | **Preeclampsia** | **25499681** |

The search in the HMDD v3.2 (<https://www.cuilab.cn/hmdd>) was done using the disease name "Preeclampsia". Members of miR-17-92 and miR-106a-363 clusters are in bold.

**Table S3 Candidate miRNA-lncRNA interactions**

| name | mirAccession | geneName | targetSites | bioComplex | clipReadNum |  |
| --- | --- | --- | --- | --- | --- | --- |
| hsa-miR-17-5p | MIMAT0000070 | HOTAIR | 1 | 6 | 92 | miRNA 3'-gatggacgtgaCATTCGTGAAAc-5'  \|\|\| \|\|\|\|\|\|\|  ncRNA 5'-acattgggtagGTATGCACTTTg-3' |
| hsa-miR-20a-5p | MIMAT0000075 | HOTAIR | 1 | 6 | 92 | miRNA 3'-gatggacgtgaTATTCGTGAAAt-5'  :\|\| \|\|\|\|\|\|\|  ncRNA 5'-acattgggtagGTATGCACTTTg-3' |
| hsa-miR-20b-5p | MIMAT0001413 | HOTAIR | 1 | 6 | 92 | miRNA 3'-gaTGGACGTGATAC-T-CGTGAAAc-5'  \|\| \| : \|\| \| \| \|\|\|\|\|\|\|  ncRNA 5'-atACATTGGGTAGGTATGCACTTTg-3' |
| hsa-miR-106a-5p | MIMAT0000103 | HOTAIR | 1 | 6 | 92 | miRNA 3'-gatggacgtgaCATTCGTGAAAa-5'  \|\|\| \|\|\|\|\|\|\|  ncRNA 5'-acattgggtagGTATGCACTTTg-3' |
| hsa-miR-17-5p | MIMAT0000070 | MALAT1 | 1 | 7 | 11 | miRNA 3'-gaTGGACGTGACATTCGTGAAAc-5'  \|: \| \|\| \| :\|\|\|\|\|\|\|  ncRNA 5'-aaATATCAACCAT-GGCACTTTc-3' |
| [hsa-miR-20a-5p](http://starbase.sysu.edu.cn/starbase2/viewMatureMirInfo.php?table=mirLncRNAInteractionsAll&database=hg19&name=hsa-miR-20a-5p) | MIMAT0000075 | [MALAT1](http://starbase.sysu.edu.cn/starbase2/viewGeneInfo.php?table=mirLncRNAInteractionsAll&database=hg19&name=MALAT1) | 1 | 7 | 11 | miRNA 3'-gaTGGACGTGATATTCGTGAAAt-5'  \|: \| \|\| \|\| :\|\|\|\|\|\|\|  ncRNA 5'-aaATATCAACCAT-GGCACTTTc-3' |
| [hsa-miR-363-3p](http://starbase.sysu.edu.cn/starbase2/viewMatureMirInfo.php?table=mirLncRNAInteractionsAll&database=hg19&name=hsa-miR-363-3p) | MIMAT0000707 | [MALAT1](http://starbase.sysu.edu.cn/starbase2/viewGeneInfo.php?table=mirLncRNAInteractionsAll&database=hg19&name=MALAT1) | 1 | 8 | 2984 | miRNA 3'-atgTCTACCTATGGCACGTTAa-5'  :\|\| \|\|\| \|\|\|\|\|\|\|  ncRNA 5'-ccaGGAAGGAGCGAGTGCAATt-3' |
| [hsa-miR-20b-5p](http://starbase.sysu.edu.cn/starbase2/viewMatureMirInfo.php?table=mirLncRNAInteractionsAll&database=hg19&name=hsa-miR-20b-5p) | MIMAT0001413 | [MALAT1](http://starbase.sysu.edu.cn/starbase2/viewGeneInfo.php?table=mirLncRNAInteractionsAll&database=hg19&name=MALAT1) | 1 | 7 | 11 | miRNA 3'-gaTGGACGTGATACTCGTGAAAc-5'  \|: \| \|\| \|\|\| \|\|\|\|\|\|\|  ncRNA 5'-aaATATCAACCATG-GCACTTTc-3' |
| [hsa-miR-106a-5p](http://starbase.sysu.edu.cn/starbase2/viewMatureMirInfo.php?table=mirLncRNAInteractionsAll&database=hg19&name=hsa-miR-106a-5p) | MIMAT0000103 | [MALAT1](http://starbase.sysu.edu.cn/starbase2/viewGeneInfo.php?table=mirLncRNAInteractionsAll&database=hg19&name=MALAT1) | 1 | 7 | 11 | miRNA 3'-gaTGGACGTGACATTCGTGAAAa-5'  \|: \| \|\| \| :\|\|\|\|\|\|\|  ncRNA 5'-aaATATCAACCAT-GGCACTTTc-3' |

The starBase platform (<http://starbase.sysu.edu.cn/>) was used.
